# Intravenous *Vipera berus* Venom-Specific Fab Fragments and Intramuscular *Vipera ammodytes* Venom-Specific F(ab’)_2_ Fragments in *Vipera* *ammodytes*-Envenomed Patients

**DOI:** 10.3390/toxins13040279

**Published:** 2021-04-14

**Authors:** Tihana Kurtović, Svjetlana Karabuva, Damjan Grenc, Mojca Dobaja Borak, Igor Križaj, Boris Lukšić, Beata Halassy, Miran Brvar

**Affiliations:** 1 Centre for Research and Knowledge Transfer in Biotechnology, University of Zagreb, Rockefellerova 10, 10000 Zagreb, Croatia; tkurtovi@unizg.hr; 2 Center of Excellence for Virus Immunology and Vaccines, CERVirVac, Rockefellerova 10, 10000 Zagreb, Croatia; 3 Clinical Department of Infectious Diseases, University Hospital of Split, Šoltanska 1, 21000 Split, Croatia; svjetlana.karabuva@gmail.com (S.K.); boris.luksic1@st.t-com.hr (B.L.); 4 School of Medicine, University of Split, Šoltanska 2, 21000 Split, Croatia; 5 Centre for Clinical Toxicology and Pharmacology, University Medical Centre Ljubljana, Zaloška cesta 7, 1000 Ljubljana, Slovenia; damjan.grenc@kclj.si (D.G.); mojca.dobaja@kclj.si (M.D.B.); 6 Department of Molecular and Biomedical Sciences, Jožef Stefan Institute, Jamova 39, 1000 Ljubljana, Slovenia; igor.krizaj@ijs.si; 7 Centre for Clinical Physiology, Faculty of Medicine, University of Ljubljana, Zaloška cesta 4, 1000 Ljubljana, Slovenia

**Keywords:** *V. ammodytes*, nose-horned viper, ViperaTAb, European viper venom antiserum, “Zagreb” antivenom, Fab fragments, F(ab’)_2_ fragments, pharmacokinetics

## Abstract

*Vipera ammodytes* (*V. ammodytes*) is the most venomous European viper. The aim of this study was to compare the clinical efficacy and pharmacokinetic values of intravenous *Vipera berus* venom-specific (paraspecific) Fab fragments (ViperaTAb) and intramuscular *V. ammodytes* venom-specific F(ab’)_2_ fragments (European viper venom antiserum, also called “Zagreb” antivenom) in *V.* *ammodytes*-envenomed patients. This was a prospective study of *V.* *ammodytes*-envenomed patients that were treated intravenously with ViperaTAb or intramuscularly with European viper venom antiserum that was feasible only due to the unique situation of an antivenom shortage. The highest venom concentration, survival, length of hospital stay and adverse reactions did not differ between the groups. Patients treated with intravenous Fab fragments were sicker, with significantly more rhabdomyolysis and neurotoxicity. The kinetics of Fab fragments after one or more intravenous applications matched better with the venom concentration in the early phase of envenomation compared to F(ab’)_2_ fragments that were given intramuscularly only on admission. F(ab’)_2_ fragments given intramuscularly had 25-fold longer apparent total body clearance and 14-fold longer elimination half-time compared to Fab fragments given intravenously (2 weeks vs. 24 h, respectively). In *V.* *ammodytes*-envenomed patients, the intramuscular use of specific F(ab’)_2_ fragments resulted in a slow rise of antivenom serum concentration that demanded their early administration but without the need for additional doses for complete resolution of all clinical signs of envenomation. Intravenous use of paraspecific Fab fragments resulted in the immediate rise of antivenom serum concentration that enabled their use according to the clinical progress, but multiple doses might be needed for efficient therapy of thrombocytopenia due to venom recurrence, while the progression of rhabdomyolysis and neurotoxic effects of the venom could not be prevented.

## 1. Introduction

**Figure 1:**
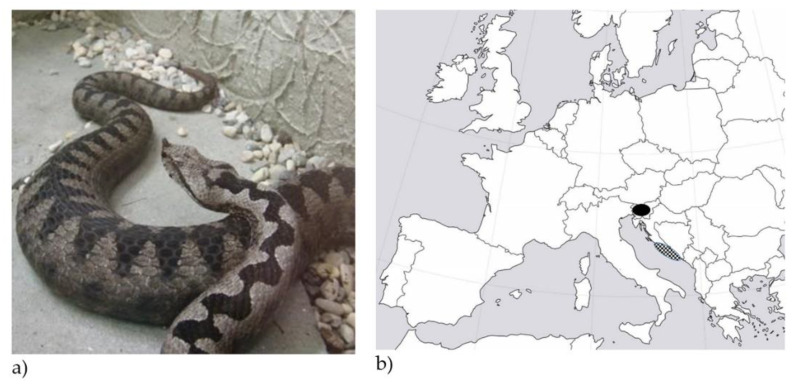
Nose-horned viper (*V. ammodytes*) (photo: M. Brvar) (**a**), which inhabits the continental and coastal areas of Slovenia (black) and Croatia (dotted) (**b**).

Snake envenoming is a rather uncommon but potentially severe injury in Europe, which is estimated to affect 0.4 to 1.1 people per 100,000 population per year [1,2]. *Vipera ammodytes* (*V. ammodytes* or nose-horned viper, Figure 1a) is the most dangerous of the European vipers due to its large size (up to 95 cm), long fangs (up to 13 mm) and high venom toxicity induced by proteolytic, haemorrhagic and neurotoxic components [3]. It inhabits southern Europe, mainly the Balkans peninsula, including coastal and central areas of Slovenia and Croatia (Figure 1b) [1].

The clinical signs of *V. ammodytes* bites include extensive local oedema and bruising, and systemic signs [1]. Neurological signs develop in 15% of *V. ammodytes* bite cases due to ammodytoxins (Atxs), the main neurotoxins of *V. ammodytes* venom [4].

Before 2015, *V. ammodytes* snakebites were successfully treated with intravenous Viperfav (Aventis Pasteur, MSD, Lyon, France), which is a formulation containing polyvalent equine F(ab’)_2_ fragments as an active principle that is raised against the venoms of *Vipera aspis*, *V. berus* and *V. ammodytes* [5], or intramuscular European viper venom antiserum (“Zagreb” antivenom) (Institute of Immunology Inc., Zagreb, Croatia), which is a formulation containing monospecific equine F(ab’)_2_ fragments against *V. ammodytes* venom [6,7]. However, due to a shortage in Viperfav and “Zagreb” antivenom availability in Slovenia between 2015 and 2019, *V. ammodytes* venomous bites were treated with ViperaTAb (MicroPharm Limited, Newcastle Emlyn, United Kingdom), which is a pharmaceutical formulation containing monospecific ovine Fab fragments against the venom of *V. berus* [8]. On the other hand, “Zagreb” antivenom was continuously available and used in Croatia, since Agency for medicinal products and medical devices of Croatia extended the expiration date of antivenom produced in 2015 until November 2019.

The protection efficacy of paraspecific *V. berus* antivenom ViperaTAb (*V. berus*-venom specific) in *V. ammodytes* venomous bites might be insufficient since it was recently shown that the proteome of *V. berus* venom is much less complex than the venom of *V. ammodytes* [4]. In particular, it contains lower levels of snake c-type lectin-like proteins (snaclecs) and no Atxs, namely, neurotoxic secreted phospholipases A_2_. Atxs are responsible for the most characteristic feature of *V. ammodytes* venom envenoming, namely, the induction of neurotoxic signs in patients, while snaclecs are probably responsible for thrombocytopenia [4]. In addition to their specificity for different viper venoms, ViperaTAb and “Zagreb” antivenom differ in pharmacokinetic profiles since ViperaTAb contains intravenous Fab fragments and “Zagreb” antivenom contains intramuscular F(ab’)_2_ fragments. In vitro immunological experiments revealed that *V. berus* antivenom ViperaTAb exhibits substantial cross-reactivity with the venoms of other *Vipera* snake species, including *V. ammodytes* [8]. An in vivo preclinical efficacy study demonstrated that ViperaTAb reduces the lethality induced by *V. ammodytes* venom, with a potency (expressed as protective efficacy) above the minimum specified by the British (and European) Pharmacopeia, thus fulfilling the regulatory requirements [8]. However, until now there has been no clinical study comparing ViperaTAb and “Zagreb” antivenom in *V. ammodytes* envenomation treatment.

The aim of this prospective study was to compare the clinical efficacy and pharmacokinetic values of intravenous paraspecific *V. berus* Fab fragments (*V. berus* venom-specific ViperaTAb) and intramuscular specific F(ab’)_2_ fragments (*V. ammodytes* venom-specific “Zagreb” antivenom) in *V. ammodytes*-envenomed patients.

## 2. Results

Over the study period, nine adult patients that met the inclusion criteria were admitted at the University Medical Centre Ljubljana and just as many at the University Hospital of Split. Their general characteristics did not differ markedly between the two centres (Table 1).

In Slovenia, five patients envenomed by *V. ammodytes* were excluded due to only minor local symptoms affecting the hands/feet that did not require antivenom therapy. In Croatia, no *V. ammodytes*-envenomed patient was excluded since all of them required antivenom treatment due to regional oedema or ecchymosis and/or systemic symptoms of envenoming. The patients treated with ViperaTAb arrived at the Emergency Department (ED) markedly later than the patients treated with “Zagreb” antivenom, median 4 vs. 1.5 h, respectively (*p* = 0.01, 95% confidence interval (CI) = −4.5, −1.0) (Table 1).

The snakebite location, serum venom concentration, symptoms and signs of envenomation on admission at the ED were similar in patients treated with ViperaTAb and “Zagreb” antivenom (Table 2).

All patients were treated with antivenom. ViperaTAb was given later than “Zagreb” antivenom (6 vs. 2.5 h, *p* = 0.03, 95% CI = −8.7, −2.7) (Table 3). The patients treated with ViperaTAb were more often given multiple doses compared to the “Zagreb” antivenom (2 vs. 1 dose) (*p* = 0.02, 95% CI = −1.3, −0.2) (Table 3) due to the further spread of oedema and recurrent thrombocytopenia. No patients had adverse reactions after the antivenom application. In Croatia, all patients treated with “Zagreb” antivenom received antihistamines and corticosteroids before admission to the ED according to the prehospital protocol. On the other hand, antiemetics were more commonly used in patients treated with ViperaTAb in Slovenia (Table 3).

Table 4 presents the most severe local and systemic symptoms and laboratory results during the envenomation and treatment. Only nausea and laboratory signs of inflammation and rhabdomyolysis were more common in patients treated with ViperaTAb after the antivenom application (Table 4). Serum myoglobin levels were higher in patients treated with ViperaTAb (148 µg/L (94–231 µg/L)) compared to the “Zagreb” antivenom (38 µg/L (37–40 µg/L)) (*p* = 0.01).

Thrombocytopenia (<150 × 10^9^) was observed in both groups (6/9 vs. 3/9) (Table 4), as well as severe thrombocytopenia (<30 × 10^9^) (4/9 vs. 1/9) (*p* = 0.13, 95% CI = 0.01, 1.8). The platelet counts increased in all patients after the ViperaTAb or “Zagreb” antivenom application, but recurrent thrombocytopenia (<30 × 10^9^) developed in only three patients treated with ViperaTAb. They were given up to two additional doses of ViperaTab to achieve a steady platelet count.

The maximum serum venom concentrations were similar. Hospital stays were non-significantly longer in patients treated with “Zagreb” antivenom compared to ViperaTAb, with 7 vs. 4 days, respectively (Table 4). No patients died.

**Figure 2:**
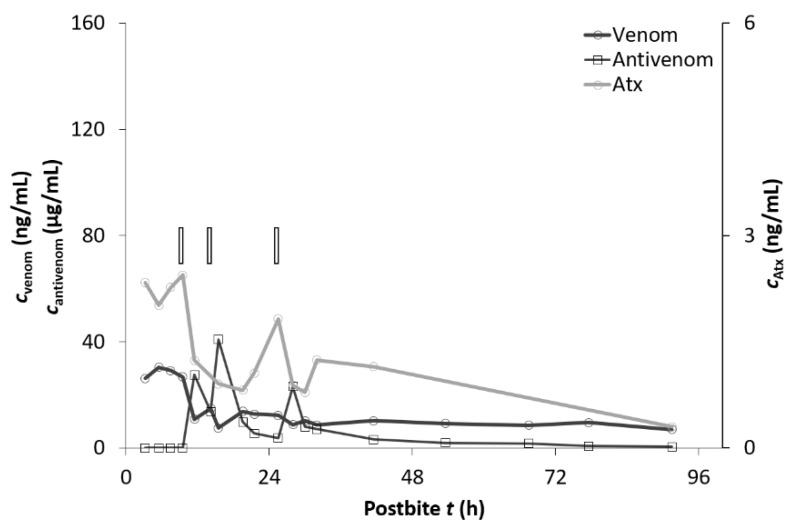
The concentration of *V. ammodytes* venom in the serum (c_venom_), serum concentrations of ammodytoxins (c_Atx_), and serum concentrations of ViperaTAb (c_antivenom_) of the representative patient (case P) bitten by *V. ammodytes* and treated with ViperaTAb (rectangle—ViperaTAb application).

Pharmacokinetic values for paraspecific ovine Fab fragments (ViperaTAb) after intravenous administration in nine patients envenomed by *V. ammodytes* are presented in Table 5 and Figure 2. The maximum serum Fab concentration in these patients was measured 2 h after the antivenom application when the first blood sample following immunotherapy was taken. However, the actual maximum serum Fab concentration would have occurred at the end of the intravenous ViperaTAb infusion.

**Figure 3:**
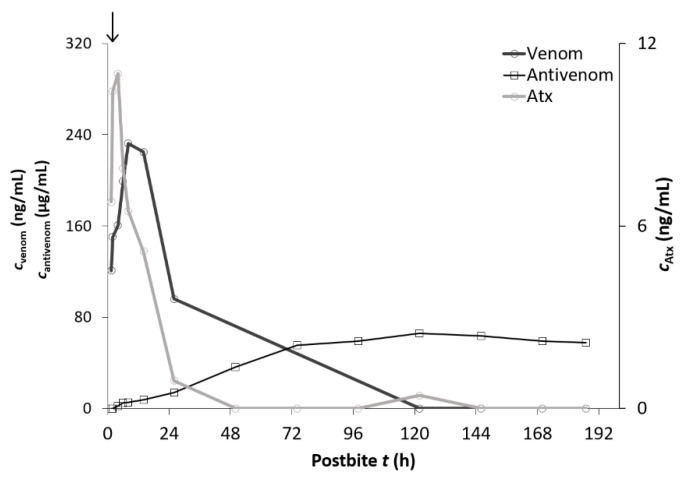
The concentration of *V. ammodytes* venom in the serum (c_venom_), serum concentrations of ammodytoxins (c_Atx_), and serum concentrations of the “Zagreb” antivenom (c_antivenom_) of the representative patient (case B) bitten by *V. ammodytes* and treated with the “Zagreb” antivenom (arrow—“Zagreb” antivenom application).

Pharmacokinetic values for specific F(ab’)_2_ fragments (“Zagreb” antivenom) after intramuscular administration were calculated in only one patient (Table 6 and Figure 3) due to the antivenom’s prolonged absorption and elimination phase and insufficient duration of sampling in the remaining eight patients. In this patient, the sampling period was 187 h after the snakebite (8 days) (Table 6 and Figure 3).

In addition, we were able to calculate time from application to maximum serum antivenom concentration (61 h (54.6–70.0 h)) and maximum serum F(ab’)_2_ concentration (74.5 μg/mL (52.4–86.3 μg/mL)) for the “Zagreb” antivenom for three patients out of nine patients treated with “Zagreb” antivenom since these three patients were discharged after the maximum serum antivenom concentration was reached. We were unable to calculate other kinetic values in these three patients since they were discharged at the beginning of the elimination phase.

**Figure 4:**
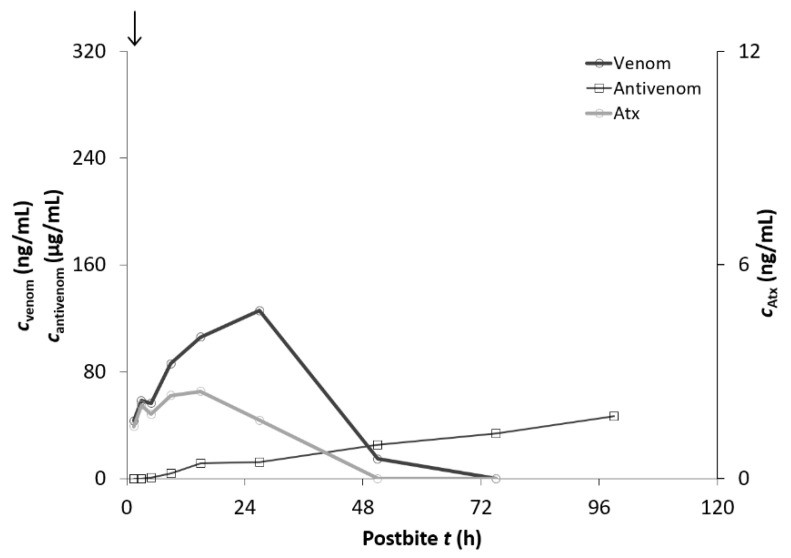
The concentration of *V. ammodytes* venom in the serum (*c*_venom_), serum concentrations of ammodytoxins (*c*_Atx_), and serum concentrations of the “Zagreb” antivenom (*c*_antivenom_) of a representative patient (case G) bitten by *V. ammodytes* and treated with “Zagreb” antivenom who was discharged before the maximum serum antivenom concentration was reached (arrow—“Zagreb” antivenom application).

The last five patients treated with “Zagreb” antivenom were discharged before the antivenom absorption was completed and the maximum serum antivenom concentration (*c*_max_) was reached due to their clinical improvement and no need for further treatment in the hospital. In these five patients, blood samples were collected up to 78–102 h after the snakebite (4 days) (Figure 4).

F(ab’)_2_ fragments in patients treated with the “Zagreb” antivenom needed a longer time to reach the maximum concentration in serum and exhibited a higher maximum serum concentration (70.3 h and 70.0 μg/mL, respectively) compared to the Fab fragments in the patients treated with ViperaTAb (2 h and 25.0 μg/mL, respectively) (*p* = 0.01, 95% CI = −86.9, −14.1 and *p* = 0.02, 95% CI = 11.5, 67.3, respectively).

## 3. Discussion

In this study, paraspecific ovine Fab fragments raised against the venom of *V. berus* (ViperaTAb) were given intravenously to *V. ammodytes*-bitten patients after the extension of oedema above a large joint or the occurrence of systemic symptoms on admission or during the observation period at the ED in Slovenia. On the other hand, specific equine F(ab’)_2_ fragments raised against the venom of *V. ammodytes* (“Zagreb” antivenom) were given intramuscularly to *V. ammodytes*-bitten patients with regional oedema or ecchymosis, and/or systemic symptoms of envenoming early after admission in Croatia. These differences were driven by an antivenom shortage and not by direct comparative data. The same route of administration was not possible according to the manufacturers. This is the first study to examine the consequences of these different practices, which was feasible only due to a unique situation of an antivenom shortage.

### 3.1. Fab and F(ab’)_2_ Pharmacokinetics Analysis

The intravenous application of ViperaTAb resulted in an immediate increase in serum Fab fragment concentration and a reciprocal decrease in serum venom concentration, except for neurotoxic Atxs. As soon as the Fab fragments started to clear from the blood, the serum concentration of venom raised again and additional doses were needed. The highest serum concentration of Fab fragments given intravenously was probably established immediately after the intravenous antivenom application and decreased with an elimination half-time of 24 h. The variable elimination half-time of the Fab fragments (9–50 h) was probably due to individual variability and irregular sampling in patients who received multiple doses since the sampling sequence was restarted after each antivenom application.

The serum concentration of F(ab’)_2_ fragments given intramuscularly was relatively low during the early stage of envenomation with a high serum venom concentration. The highest serum concentration of F(ab’)_2_ fragments was slowly and progressively reached only after 2.5–5 days, after the venom concentration had already decreased, since the venom has an apparent half-time of 8 h. No detectable levels of venom from European vipers could be expected in serum requiring neutralisation more than 72 h after a bite [9,10,11]. However, high serum antivenom concentration early after intramuscular application might not be crucial since most of the venom is most likely absorbed in the lymphatic circulation [12] and could be removed before its entrance into circulation by an antivenom released in the lymphatic system from the intramuscular depot, as it was described in coral snake envenomation [13,14]. Nevertheless, we have to consider the difference between the local effects of these two venoms since neurotoxic coral snake venom does not cause local damage, including injury of the lymphatic system with lymphedema, or a delayed uptake from tissue injury, as does *V. ammodytes* venom [13]. For a *V. ammodytes* bite, the lymphatic pathway could be effective if the antivenom is given intramuscularly since large antivenom macromolecules can be absorbed only in lymphatic capillaries with an incomplete basal lamina, and venom neutralisation can happen in larger lymphatic ducts and local lymphatic nodes before it reaches the bloodstream [15].

In addition to the slow release of F(ab’)_2_ fragments from muscle and the prolonged time to reach maximum serum concentration, F(ab’)_2_ fragments given intramuscularly had a 25-fold slower apparent total body clearance compared to Fab fragments given intravenously. Furthermore, the elimination half-time of F(ab’)_2_ fragments was almost two weeks, which was 14-fold longer compared to the elimination half-time of Fab fragments given intravenously. The beneficial side of the prolonged elimination half-time of F(ab’)_2_ fragments was the circumvention of the venom recurrence and there was no need for additional doses. As expected, the elimination half-time of F(ab’)_2_ given intramuscularly was also longer compared to the half-life of the same F(ab’)_2_ fragments given intravenously in *V. aspis* envenomation (40–100 h) [9].

In this study, the highest serum venom concentration measured during the hospitalisation did not differ between the two groups despite the use of different immunoglobulin fragments, doses, routes and timelining. It seems that the kinetics of serum Fab fragments after one or more intravenous doses matched better with the venom concentration in the early stage of envenomation compared to the F(ab’)_2_ fragments that were given intramuscularly only once early after admission. The highest serum F(ab’)_2_ fragments’ concentration after the intramuscular application was higher compared to the highest measured concentration of Fab fragments that were given intravenously, but it was reached only after 2.5–5 days. However, the actual maximum serum Fab concentration probably occurred at the end of the intravenous ViperaTAb infusion, not 2 h from the start when the first sample was taken. In the future, earlier and more frequent blood sampling should be performed in patients that are treated intravenously in order to more precisely determine the maximum serum Fab concentration and time required to reach it. In an animal study, intramuscular application of F(ab’)_2_ fragments resulted in a maximum plasma concentration that was only 10% of the intravenous dose and it was established 48 h after application [16,17]. The possible explanations for this discrepancy in our study could be the higher dose of F(ab’)_2_ fragments compared to Fab fragments despite more than half of the patients being given two or three doses of Fab fragments. A 2.5-fold larger apparent volume of distribution of Fab fragments compared to F(ab’)_2_ fragments found in this study might also be one of the reasons since it indicates a faster diffusion of Fab fragments into the extravascular compartment and a faster decrease in the serum Fab fragment concentration [17,18].

### 3.2. The Clinical Picture and Outcome of the Envenomation and Antivenom Therapy

The outcomes of both antivenom therapies, including the length of the hospital stay, survival and adverse reactions, did not differ. The local symptoms on admission were present in all patients but laboratory signs of rhabdomyolysis developed in more patients treated intravenously with Fab fragments. This could indicate more severe progress of local symptoms in patients treated with Fab fragments. However, this might also be due to the later application of antivenom in Slovenia due to the delayed admission at the ED and a longer observation period since the snakebites happened in more distant and mountainous areas; however, Slovenians might also be more hesitant to visit the ED. When interpreting the differences between the groups, we must also consider that the groups were small. The small groups and the absence of the measurement of local symptoms, such as the size of local oedema and ecchymosis and the severity of local pain, are the main limitations of this study. The number of included patients could not have been increased since *V. ammodytes* venom-specific antivenom has again become available in Slovenia and the study was finished.

Neurological symptoms that are characteristic of *V. ammodytes* envenomation appeared in 15% of patients and did not differ between the groups. However, severe neurotoxicity developed in one patient bitten by *V. ammodytes* and treated with multiple doses of paraspecific antivenom raised against *V. berus* venom. Specifically, he first developed cranial nerve palsies, including bilateral ptosis, ophthalmoplegia and dysphagia. He then developed respiratory insufficiency due to hypoventilation and aspiration pneumonia due to dysphagia. This resulted in a prolonged treatment that included mechanical ventilation (presented in Table 1, Table 2 and Table 3). On the other hand, two patients with neurological symptoms treated with specific equine F(ab’)_2_ raised against *V. ammodytes* venom did not suffer from additional complications and were discharged after 1 week (Table 1, Table 2 and Table 3). Therefore, even though paraspecific Fab fragments raised against *V. berus* were shown to be statistically equally effective as specific F(ab’)_2_ fragments raised against *V. ammodytes*, they were inefficient in the patient with cranial nerve palsies due to the lack of antibodies directed against neurotoxic Atxs. Interestingly, paraspecific Fab fragments raised against *V. berus* venom effectively reversed the thrombocytopenia despite *V. berus* venom not containing as much snaclecs as *V. ammodytes* venom [4] and there was no difference in the platelet numbers between the groups in this study. However, an increase of platelets after the application of paraspecific Fab fragments raised against *V. berus* venom was just transient and additional doses of Fab fragments were needed, which is consistent with Fab fragments’ kinetics. The same was also observed in the use of paraspecific Fab fragments raised against *V. berus* venom in patients envenomed by *V. aspis* [19]. This implies that only the use of F(ab’)_2_ fragments raised against *V. ammodytes* venom reduces the development of medically significant complications after a *V. ammodytes* snakebite, as well as the need for repetitive antivenom application.

## 4. Conclusions

In patients envenomed by *V. ammodytes,* intramuscular F(ab’)_2_ fragments raised against *V. ammodytes* venom should be used as soon as possible since intramuscular use results in a steady rise of antivenom serum concentration. Due to the prolonged persistence of the antivenom in the organism, there is no venom recurrence and no need for additional doses.

The intravenous use of Fab fragments raised against *V. berus* venom resulted in an immediate rise of the active principle serum concentration that enabled their use according to clinical progress, but multiple doses were needed for efficient therapy against thrombocytopenia due to venom recurrence, while the progression of rhabdomyolysis and neurotoxic effects of the venom could not be prevented.

In patients envenomed by *V. ammodytes*, F(ab’)_2_ fragments raised against *V. ammodytes* venom should be used, but Fab fragments raised against *V. berus* venom could give satisfactory protection in the situation of a specific antivenom shortage.

## 5. Materials and Methods

This was a prospective study of consecutive patients envenomed by *V. ammodytes* venom and treated either with ViperaTAb at the government University Medical Centre Ljubljana (Slovenia) or “Zagreb” antivenom at the University Hospital of Split (Croatia) from 2015 to 2019 following respective national snakebite guidelines.

The University Medical Centre Ljubljana is a tertiary referral centre in the Slovenian capital city of Ljubljana that serves a local population of 600,000 inhabitants and a national population of two million. The University Hospital of Split (Croatia) is a tertiary referral centre in Split that serves a local population of 1,000,000 inhabitants in the coastal area of Croatia (Dalmatia). The study was approved by the Croatian National Medical Ethics Committee and Slovenian National Medical Ethics Committee (no. 87/07/15).

### 5.1. Patients

Patients over 18 years old envenomed by a *V. ammodytes* snakebite and treated with antivenom were included in the study. Cases were only included if the *V. ammodytes* envenomation was confirmed by the detection of *V. ammodytes*-specific Atxs in the patient’s serum and/or the snake was identified in situ by photographing.

The following data were collected prospectively from the patients: age, sex, location and time of snakebite, local signs (pain, oedema, ecchymosis), systemic signs (nausea, vomiting, diarrhoea, dizziness, syncope, conscious level, cranial nerve palsies, pulse, systolic blood pressure), laboratory results (myoglobin, creatine kinase, creatine, troponin I, liver tests, international normalized ratio (prothrombin time), activated partial thromboplastin time, D-dimer, fibrinogen, platelets count, C-reactive protein, procalcitonin, leucocyte count, lactate), therapy (medication, timing, dose, adverse effects, length of stay) and outcome (death). The clinical picture and laboratory results were evaluated at the ED before the antivenom therapy and re-evaluated through the entire hospital stay. The final symptoms and laboratory results were the most severe/highest registered.

The follow-up examination of each patient was accomplished at the University Hospital of Split 2–3 weeks and 2 months after discharge. In Slovenia, the patients were instructed to return in case of additional symptoms, such as arthralgia.

Tachycardia was diagnosed in patients with a pulse above 100 bpm, hypotension in patients with systolic pressure below 90 mmHg and shock in patients who had hypotension and elevated serum lactate concentration. Cranial nerve palsies included ptosis, ophthalmoplegia or dysphagia. Acute renal injury was diagnosed in patients who had a serum creatinine level at least twice the upper normal level. Rhabdomyolyses was diagnosed in patients who had serum myoglobin and creatine kinase levels that were at least twice the upper normal level. Acute respiratory failure was diagnosed in patients with tachypnoea (>25 bpm) and partial pressure of oxygen (PaO_2_) < 8 kPa. Acute myocardial injury was determined as positive troponin I Ultra (>0.10 µg/L) or troponin I High sensitivity (>60 ng/L). Leucocytosis and thrombocytopenia were defined as leucocyte and platelets counts above 11 × 10^9^/L and below 150 × 10^9^/L, respectively. Disseminated intravascular coagulation was diagnosed in patients with thrombocytopenia, elevated D-dimer, prolonged prothrombin (international normalized ratio) and activated partial thromboplastin times and decreased fibrinogen level.

### 5.2. Antivenom Therapy

In Slovenia, the patients were given a dose of 8 mL of ViperaTAb (Fab fragments at a concentration of 25 mg/mL) diluted in 100 mL of 0.9% NaCl intravenously over 30 min. The indication for antivenom therapy was an extension of oedema above one large joint reaching arm/thigh or the occurrence of systemic signs of *V. ammodytes* envenomation. The antivenom was given at the ED or/and Centre for Clinical Toxicology and Pharmacology.

In Croatia, the patients were given a dose of 10 mL of “Zagreb” antivenom (F(ab’)_2_ fragments at a concentration of 100 mg/mL) intramuscularly following national guidelines. The indication for antivenom therapy was regional oedema or ecchymosis and/or systemic symptoms of envenoming. The antivenom was given at the Clinical Department of Infectious Disease immediately after the examination at the ED, which is part of the same department.

Supplemental doses were given using the same methods, depending on how the envenomation developed (e.g., further spread of local signs and worsening of laboratory results, especially thrombocytopenia).

The supportive therapy was given regarding the clinical presentation and laboratory results, except for antihistamines and corticosteroids, which were given to all patients before the “Zagreb” antivenom by prehospital emergency physicians during transport to the hospital ward.

### 5.3. Blood Samples

Blood samples were taken in serum tubes upon arrival at the ED and then 0, 2, 4, 6, 12 and 24 h after each antivenom application. The sampling was furtherly prolonged until discharge in 12 h intervals. Blood samples were immediately centrifuged, aliquoted and frozen at −50 °C until the venom and antivenom measurement.

### 5.4. Reagents and Chemicals

The horseradish-peroxidase-conjugated rabbit anti-guinea-pig IgG (HRP-anti-guinea-pig IgG) and horseradish-peroxidase-conjugated rabbit anti-equine IgG (HRP-anti-equine IgG) antibodies were from Bio-Rad Laboratories (Hercules, CA, USA). The goat anti-equine F(ab’)_2_ antibody conjugated with horseradish peroxidase (HRP-anti-equine F(ab’)_2_ and horseradish-peroxidase-conjugated rabbit anti-ovine IgG antibody (HRP-anti-ovine IgG) were from Antibodies Online (Aachen, Germany). Bovine serum albumin (BSA), Tween 20 and *o*-phenylenediamine dihydrochloride (OPD) were from Sigma-Aldrich (St. Louis, MO, USA). Chemicals for the buffers and solutions were from Kemika, Croatia.

*V. ammodytes* venom and recombinant ammodytoxin A (AtxA), which were used as standards in the respective ELISA assays, were supplied by the Institute of Immunology Inc., Croatia, or produced as described in Liang et al. [20], respectively.

ViperaTAb was supplied by MicroPharm Ltd., Newcastle Emlyn, U.K. “Zagreb” antivenom was supplied by the Institute of Immunology Inc., Zagreb, Croatia.

### 5.5. Quantification of V. ammodytes Venom in Sera Samples

A microtitre plate was coated with in-house rabbit anti-*V. ammodytes* venom IgG (5 μg/mL) in 0.05 M carbonate buffer, pH 9.6 (100 μL/well), and left overnight at room temperature (RT). After washing and blocking with 2% (m/v) BSA in a PBS buffer comprising 0.05% (*v/v*) Tween 20 (200 μL/well) for 2 h at 37 °C, the investigated sera (in suitable dilution, depending on the patient) were added in duplicates and incubated overnight at RT.

The whole venom solution, which was used as a standard, was added to eight serial twofold dilutions, starting from 100 ng/mL and prepared in respective matrixes in duplicates (100 μL/well). A pool of sera from unbitten individuals (analysed in the same dilution as the investigated sera) was used as a negative control. The plate was extensively washed and incubated first with in-house horse anti-*V. ammodytes* venom IgG (100 μL/well of 5.7 μg/mL) and then with HRP-anti-equine IgG (100 μL/well of 4000-fold dilution). Finally, after washing, the OPD solution (5.5 mM in 0.15 M citrate-phosphate buffer, pH 5.0) with 30% (*v/v*) H_2_O_2_ (0.5 μL/mL of OPD solution) was added and incubated for half an hour at RT in the dark. The enzymatic reaction was stopped with 1 M H_2_SO_4_ (50 μL/well) and the absorbance at 492 nm was measured. The venom content was determined by multiplying each concentration, which was obtained from the standard curve using the corresponding dilution factor.

### 5.6. Quantification of Atxs in Sera Samples

The ELISA for determination of Atxs was performed in a similar manner to the whole venom with few exceptions. The coating was done with in-house rabbit anti-Atx IgG (100 μL/well of 1 μg/mL). The investigated sera (in suitable dilution, depending on the patient) and pure AtxA solution as a standard (eight serial twofold dilutions starting from 10 ng/mL in each respective matrix) were added in duplicates after washing and blocking. After washing, the plates were incubated first with in-house guinea pig anti-AtxA IgG (100 μL/well of 0.3 μg/mL) and then with HRP-anti-guinea pig IgG (100 μL/well with a 10,000-fold dilution). The final steps were performed as described in the previous section.

### 5.7. Quantification of Antivenom in Sera Samples

The ELISA for the determination of antivenom IgG fragments, namely, Fab or F(ab’)_2_, was performed as follows. A microtitre plate was coated with *V. ammodytes* venom (1 μg/mL) in a 0.05 M carbonate buffer, pH 9.6 (100 μL/well), and left overnight at RT. After washing and blocking the wells with 0.5% (*m*/*v*) BSA in a PBS buffer comprising 0.05% (*v/v*) Tween 20 (200 μL/well) for 2 h at 37 °C, the investigated sera were added in a suitable range of twofold dilutions in duplicates (100 μL/well), as well as ViperaTAb (starting from 100 ng/mL) or “Zagreb” antivenom (starting from 100 ng/mL), which were used as standards for the Fab and F(ab’)_2_ fragments’ quantification, respectively. The incubation was performed overnight at RT. The plate was extensively washed and incubated with HRP-anti-ovine IgG (100 μL/well with a 5000-fold dilution) for Fab or HRP-anti-equine F(ab’)_2_ IgG (100 μL/well with a 25,000-fold dilution) for the F(ab’)_2_ fragments’ quantification. The final steps were performed as described above.

### 5.8. Pharmacokinetic Analysis

Pharmacokinetic analysis of the measured concentrations was performed using PKSolver add-in software (version 2.0, China Pharmaceutical University, Nanjing, China) for Microsoft Excel [21]. Noncompartmental analysis of the data after extravascular or intravenous constant infusion input was performed for the calculation of the pharmacokinetic values.

### 5.9. Statistical Analysis

Data are presented as median (interquartile range (IQR)) for continuous variables and the frequency (percentage) for categorical variables. Odds ratios (ORs) with the Haldane–Anscombe correction and the corresponding confidence interval are presented for categorical variables. Confidence intervals (CIs) for the difference between means of continuous variables with a significant difference between groups are presented. Multivariate analysis was performed using logistic regression modelling. The Mann–Whitney test was used to identify the pharmacokinetic differences between antivenoms. A *p*-value of 0.05 was considered significant. Analyses were carried out with the IBM SPSS Statistics for Windows, Version 23.0., Armonk, NY, U.S.A.

## Figures and Tables

**Table 1:** General characteristics of the *V. ammodytes*-envenomed patients.

General Characteristics of the *V. ammodytes* Envenomed Patients	ViperaTAb (*n* = 9 Patients)	“Zagreb” Antivenom (*n* = 9 Patients)	*p*
Age (median, IQR) (year)	40 (35–60)	58 (36–67)	0.44
Gender (male)	8/9	7/9	1.00
Weight (median, IQR) (kg)	72 (70–75)	93 (81–98)	0.11
Comorbidities	4/9	4/9	0.34
Distance from the scene to the ED (median, IQR) (km)	69 (61–98)	44 (40–50)	0.09
Time from bite to admission at the ED (median, IQR) (h)	4 (3–4)	1.5 (1.5–1.75)	0.01

Legend: ED—emergency department; IQR—interquartile range.

**Table 2:** *V. ammodytes*-envenomed patients’ characteristics before the antivenom application.

Symptoms and Laboratory Results	ViperaTAb (*n* = 9 Patients)	“Zagreb” Antivenom (*n* = 9 Patients)	OR (95% CI)	*p*
Bite location				
Arm	7/9	7/9	1.0 (0.11–9.23)	1.00
Leg	2/9	2/9	1.0 (0.11–9.23)	1.00
Venom concentration (median, IQR) (ng/mL)	52.5 (27.3–106.3)	47.3 (37.8–90.3)	NA	0.65
Atx concentration (median, IQR) (ng/mL)	2.3 (1.6–5.9)	3.2 (1.8–5.1)	NA	0.85
Local pain	9/9	9/9	1.0 (0.02–55.80)	1.00
Local oedema	9/9	9/9	1.0 (0.02–55.80)	1.00
Ecchymosis	4/9	9/9	23.22 (1.04–517.96)	0.13
Nausea	5/9	2/9	0.23 (0.03–1.77)	0.13
Vomiting	3/9	1/9	0.25 (0.02–3.04)	0.13
Dizziness	4/9	0/9	0.06 (0.01–1.44)	0.01
Syncope	1/9	0/9	0.29 (0.01–8.34)	0.13
Cranial nerve palsies	1/9	2/9	0.30 (0.01–8.35)	1.00
Tachycardia	5/9	2/9	0.23 (0.03–1.77)	0.57
Hypotension	3/9	0/9	0.10 (0.01–2.23)	0.02
Shock	1/9	0/9	0.30 (0.01–8.35)	0.13
Acute respiratory failure	1/9	0/9	0.30 (0.01–8.35)	0.13
Rhabdomyolysis	5/9	1/9	0.10 (0.01–1.17)	0.13
Acute renal failure	0/9	0/9	1.00 (0.02–55.80)	1.00
Acute myocardial injury	1/9	0/9	0.30 (0.01–8.35)	0.13
Disseminated intravascular coagulation	0/9	0/9	1.00 (0.02–55.80)	1.00
D-dimer (>500 µg/L)	7/9	4/9	0.23 (0.03–1.77)	0.06
International normalized ratio (INR) (>1.3)	2/9	0/9	0.16 (0.01–3.81)	0.13
Activated partial thromboplastin time (>36 s)	2/9	0/9	0.16 (0.01–3.81)	0.13
Fibrinogen (<1.8 g/L)	0/9	0/9	1.00 (0.02–55.80)	1.00
Thrombocytopenia (<150 × 10^9^)	6/9	3/9	0.25 (0.04–1.77)	0.57
Procalcitonin (>0.24 µg/L)	2/9	0/9	0.16 (0.01–3.81)	0.02
Leucocytosis (>11 × 10^9^/L)	7/9	4/9	0.23 (0.03–1.77)	0.13

Legend: Atxs—ammodytoxins; CI—confidence interval; IQR—interquartile range; NA—not available; OR—odds ratio.

**Table 3:** Therapy of *V. ammodytes*-envenomed patients.

Therapy	ViperaTAb (*n* = 9 Patients)	“Zagreb” Antivenom (*n* = 9 Patients)	OR (95% CI)	*p*
**Antivenom Therapy**				
Time from bite to first dose (median, IQR) (h)	6 (4.5–10)	2.5 (2–3)	NA	0.03
Time from admission at the ED to first dose (median, IQR) (h)	2 (1.5–6.5)	1 (0.5–1.5)	NA	0.13
Multiple doses	5/9	0/9	0.04 (0.01–0.96)	0.02
Number of doses (median, IQR)	2 (1–2)	1 (1–1)	NA	0.03
Anaphylactic reaction	0/9	0/9	1.00 (0.02–55.80)	1.00
Serum sickness	0/9	0/9	1.00 (0.02–55.80)	1.00
**Other Therapy**				
Corticosteroids	1/9	9/9	107.66 (3.85–3013.31)	0.01
Antihistamines	2/9	9/9	57.00 (2.36–1375.85)	0.02
Analgesics	7/9	3/9	0.14 (0.02–1.16)	0.02
Antiemetics	8/9	1/9	0.02 (0.01–0.30)	0.01
Antibiotics	1/9	4/9	6.40 (0.55–78.89)	1.00
Low-molecular-weight heparin	1/9	0/9	0.30 (0.01–8.35)	0.13
Platelet transfusion	1/9	0/9	0.30 (0.01–8.35)	0.13
Red blood cell transfusion	1/9	0/9	0.30 (0.01–8.35)	0.13
Oxygen	2/9	0/9	0.16 (0.01–3.81)	0.13
Mechanical ventilation	1/9	0/9	0.30 (0.01–8.35)	0.13
Noradrenaline infusion	1/9	0/9	0.30 (0.01–8.35)	0.13

Legend: CI—confidence interval; IQR—interquartile range; NA—not available; OR—odds ratio.

**Table 4:** The most severe symptoms and maximum laboratory results during the envenomation and antivenom therapy in *V. ammodytes*-envenomed patients.

Symptoms and Laboratory Results	ViperaTAb (*n* = 9 Patients)	“Zagreb” Antivenom (*n* = 9 Patients)	OR (95% CI)	*p*
Maximum venom concentration (median, IQR) (ng/mL)	52.5 (29.7–106.3)	84.0 (47.3–125.7)	NA	0.36
Maximum Atx concentration (median, IQR) (ng/mL)	2.4 (1.6–6.0)	3.8 (2.7–8.3)	NA	0.65
Time from bite to maximum venom concentration (median, IQR) (h)	5.5 (4–7)	6.5 (1.5–8)	NA	0.22
Local pain	9/9	9/9	1.00 (0.02–55.80)	1.00
Local oedema	9/9	9/9	1.00 (0.02–55.80)	1.00
Local lymphadenitis	8/9	4/9	0.10 (0.01–1.17)	0.13
Oedema spread to trunk	4/9	4/9	1.00 (0.16–6.42)	0.34
Ecchymosis	5/9	9/9	15.5 (0.70–346.74)	0.13
Nausea	7/9	2/9	0.08 (0.01–0.75)	0.02
Vomiting	3/9	1/9	0.25 (0.02–3.04)	0.13
Hypotension	4/9	0/9	0.06 (0.01–1.43)	0.01
Shock	1/9	0/9	0.30 (0.01–8.35)	0.13
Tachycardia	5/9	1/9	0.10 (0.01–1.17)	0.13
Dizziness	4/9	0/9	0.06 (0.01–1.43)	0.01
Syncope	1/9	0/9	0.30 (0.01–8.35)	0.13
Cranial nerve palsies	1/9	2/9	2.29 (0.17–30.96)	1.00
Acute respiratory failure	2/9	0/9	0.16 (0.01–3.81)	0.13
Acute renal failure	0/9	0/9	1.00 (0.02–55.80)	1.00
Rhabdomyolysis	7/9	1/9	0.04 (0.01–0.48)	0.02
Acute myocardial injury	1/9	0/9	0.30 (0.01–8.35)	0.13
Disseminated intravascular coagulation	1/9	0/9	0.30 (0.01–8.35)	0.13
Thrombocytopenia (<150 × 10^9^)	6/9	3/9	0.25 (0.04–1.77)	0.57
D-dimer (>500 µg/L)	9/9	5/9	0.06 (0.01–1.44)	0.13
International normalized ratio (INR) (>1.3)	4/9	0/9	0.06 (0.01–1.43)	0.02
Activated partial thromboplastin time (>36 s)	3/9	0/9	0.10 (0.01–2.23)	0.13
Fibrinogen (<1.8 g/L)	1/9	0/9	0.30 (0.01–8.35)	0.13
Procalcitonin (>0.24 µg/L)	6/9	0/9	0.03 (0.01–0.65)	0.01
Leucocytosis (>11 × 10^9^/L)	8/9	6/9	0.25 (0.02–3.04)	0.26
Length of hospital stay (median, IQR, days)	4 (2–9)	7 (4–7)	NA	0.14
Death	0/9	0/9	1.00 (0.02–55.80)	1.00

Legend: Atxs—ammodytoxins; CI—confidence interval; IQR—interquartile range; NA—not available; OR—odds ratio.

**Table 5:** Pharmacokinetic values of *V. berus* venom-specific ovine Fab fragments (ViperaTAb) after intravenous administration in the patients envenomed by *V. ammodytes*.

Pharmacokinetic Values	Intravenous ViperaTAb (*n* = 11 Doses)
*t*_max_ (h)	2 (2–2.3)
*c*_max_ (μg/mL)	25.0 (23.3–33.8)
*t*_1/2_ (h)	24.5 (9.0–50.0)
AUC_∞_ (μg·h/mL)	250.3 (218.9–326.0
*V*_ss_ (mL/kg)	317.5 (182.3–415.1)
*V*_z_ (mL/kg)	473.1 (230.0–651.5)
MRT (h)	22.1 (11.4–49.9)
CL (mL/h·kg)	9.8 (8.9–13.3)

Legend: AUC_∞_, area under the serum concentration–time curve from time zero to infinity; CL, apparent total body clearance of the drug from serum; *c*_max_, maximum (peak) serum drug concentration; *t*_1/2_, half-life; MRT, mean residence time; *t*_max_, time to reach maximum (peak) serum concentration following the drug administration; *V*_ss_, steady-state volume of distribution; *V*_z_, apparent volume of distribution during the terminal phase. Data are presented as median (interquartile range (IQR)).

**Table 6:** Pharmacokinetic values of *V. ammodytes* venom-specific equine F(ab’)_2_ fragments (“Zagreb” antivenom) after intramuscular administration in the patient envenomed by *V. ammodytes*.

Pharmacokinetic Values	Intramuscular “Zagreb” Antivenom (*n* = 1 Dose)
*t*_max_ (h)	120.0
*c*_max_ (μg/mL)	66.1
*t*_1/2_ (h)	317.2
AUC_∞_ (μg·h/mL)	3.5 × 10^4^
*V*_z_ (mL/kg)	190.9
MRT (h)	510.2
CL (mL/h·kg)	0.4

Legend: AUC_∞_, area under the serum concentration–time curve from time zero to infinity; CL, apparent total body clearance of the drug from serum; *c*_max_, maximum (peak) serum antivenom concentration; MRT, mean residence time; *t*_1/2_, elimination half-life; *t*_max_, time to reach maximum (peak) serum concentration following the antivenom administration; *V*_z_, apparent volume of distribution during the terminal phase.

## Data Availability

Data is contained within the article.
